# Tumor-Suppressive Function of lncRNA-MEG3 in Glioma Cells by Regulating miR-6088/SMARCB1 Axis

**DOI:** 10.1155/2020/4309161

**Published:** 2020-01-11

**Authors:** Xin Gong, Meng-Yi Huang

**Affiliations:** Department of Neurosurgery, Hunan Provincial People's Hospital, The First Affiliated Hospital of Hunan Normal University, Changsha, Hunan 410005, China

## Abstract

**Objective:**

Mounting evidence has elaborated the implication of long noncoding RNAs (lncRNAs) in tumorigenesis of several cancers, including glioma. However, little was known about the mechanism of lncRNA maternally expressed gene 3 (MEG3) in the development and progression of glioma. This work is designed to explore the effect of MEG3 on glioma progression and its possible mechanism.

**Methods:**

Expressions of lncRNA-MEG3 and SMARCB1 were detected in human glioblastoma U87 and U251 cell lines. Gain and loss of function of MEG3 or/and miR-6088 was performed in U87 and U251 cells to observe its effect on cell proliferation and migration as well as on epithelial-mesenchymal transition (EMT) related markers. Luciferase reporter gene assay was employed to inspect the interactions among MEG3, miR-6088, and SMARCB1.

**Results:**

MEG3 and SMARCB1 expressions were downregulated in glioma cells. Transfection of pcDNA3.1-MEG3 or pcDNA3.1-SMARCB1 plasmids could clearly block cell proliferation, migration, and EMT progression. MEG3 functions as a sponge for miR-6088, while SMARCB1 is a downstream protein of miR-6088. Transfection of miR-6088 mimic or si-SMARCB1 could obviously reverse the favorable effect of pcDNA3.1-MEG3 on glioma progression.

**Conclusion:**

Collectively, the evidence in this study indicated that MEG3 was downregulated in glioma cells and inhibited proliferation and migration of glioma cells via regulating miR-6088/SMARCB1 axis.

## 1. Introduction

Glioma, a malignant tumor, is the most common intracranial primary cancer with the highest morbidity and mortality rates worldwide [1–4]. In spite of the great efforts on the clinical development, the long-term prognosis and postoperative outcomes for patients are still far from being satisfactory [5, 6]. Moreover, palliative therapies fail to achieve the desirable therapeutic efficiency in consideration of the vague understanding on the potential pathophysiological mechanisms of glioma progression [7]. Therefore, it is of great clinical value to further explore the detailed pathogenic mechanism of glioma progression and therefore to identify more effective diagnostic strategies and potential therapeutic targets.

Long noncoding RNAs (lncRNAs) are a subset of RNAs that exceed 200 nucleotides in length with limited or no protein-coding ability [8]. The dysregulation of lncRNAs in glioma has been revealed. For example, lncRNA MALAT1 enhances the activity and proliferation ability of glioma stem cells and promotes glioma tumorigenesis [9]. LncRNA maternally expressed gene 3 (MEG3), located on human chromosome 14q32.3, is a tumor suppressor gene [10]. Also, a study proved that lncRNA MEG3 could regulate tumorigenesis through its interaction with microRNA [11]. For example, lncRNA MEG3 inhibits the tumorigenesis of hemangioma through sponging miR-494 and mediating PTEN/PI3K/AKT pathway [12]. However, the roles of lncRNA MEG3 in glioma development and its molecular mechanisms remain unclear.

SMARCB1 is also known as INI1, whose downregulation is associated with aggressive behavior of glioblastoma [13]. Also, a report has revealed that SMARCB1 directly blocks transcription of glioma-associated oncogene homologue (GLI), thereby decreasing the downstream hedgehog pathway target genes like GL1, GL2, and protein patched homologue 1 [14]. However, it remains to be explored whether SMARCB1 implicated in the proliferation and migration of glioma cells. In this work, we found downregulated MEG3 and SMARCB1 in glioma cells, but no direct interaction of MEG3 and SMARCB1 was identified. Therefore, we aim to explore the possible role of MEG3 and SMARCB1 in glioma cells and to further clarify the mechanism herein. The application of dual-luciferase reporter gene assay and gain and loss of function found that MEG3 serves in glioma cells as a competitive endogenous RNA (ceRNA). Altogether, the potential mechanism herein is that MEG3 negatively targets miR-6088 to regulate SMARCB, thus mediating the proliferation and migration of glioma cells.

## 2. Materials and Methods

### 2.1. Cell Culture

Normal human astrocytes (NHA) and human glioblastoma U251 and U87 cells purchased from the American Type Culture Collection (ATCC) cell bank were maintained in DMEM (Thermo Fisher Scientific, Wilmington, DE, USA) with 10% Fetal Bovine Serum (FBS) (Thermo Fisher Scientific, Wilmington, DE, USA) and cultured in a humid atmosphere of 5% CO_2_ at 37°C.

### 2.2. Cell Transfection

U251 and U87 cells in logarithmic phase were transfected with 2 ug of pcDNA3.1, pcDNA3.1-MEG3, si-NC, si-MEG3, pcDNA3.1-SMARCB1, si-MEG3, 100 nM mimic NC, miR-6088 mimic, inhibitor NC, or miR-6088 inhibitor plasmids (RiboBio Co., Ltd, Guangzhou, China) and correspondingly grouped into pcDNA3.1 group, pcDNA3.1-MEG3 group, si-NC group, si-MEG3 group, pcDNA3.1-SMARCB1 group, si-SMARCB1 group, mimic NC group, miR-6088 mimic group, inhibitor NC group, miR-6088 inhibitor group, si-MEG3 + inhibitor NC group, si-MEG3 + miR-6088 inhibitor group, si-MEG3 + pcDNA3.1 group, and si-MEG3 + pcDNA3.1-SMARCB1 group. All transfections were performed in strict accordance with Lipofectamine 2000 reagent instructions (Thermo Fisher Scientific, MA, USA). The transfected cells were cultured with serum-free DMEM and incubated in 5% CO_2_ at 37°C constant temperature incubator.

### 2.3. MTT Assay

Cells were counted after corresponding treatment for 24 h, 48 h, 72 h, and 96 h, respectively. Cell suspension (100 *μ*L, 10^4^ to 10^5^ cells) placed in a 96-well plate was incubated at 37°C in atmosphere of 5% CO_2_ in air. Three duplicate wells were set for each group. Then 20 *μ*L of MTT solution (5 mg/mL, Sigma, USA) was added for further incubation at 37°C with 5% CO_2_. After 4 h of incubation, the culture medium was removed and 150 *μ*L of DMSO was then added to each well. The culture medium was shaken gently for 10 min to promote the solubility of crystallization. The absorbance value (OD_570_ value) of each well was tested at wavelength of 540 nm on the enzyme-linked immunometric meter. The absorbance value was set as ordinate and time as abscissa to draw MTT curve. The absorbance value of each group was measured for 3 times to obtain the average data.

### 2.4. Colony Formation Assay

After digested by 0.25% trypsinization, the monolayer cells in logarithmic phase were suspended in 10% FBS culture medium. Next, cell suspension at density of 50, 100, and 200 per cell was separately inoculated in 10 ml culture medium at 37°C by gently shaking till cells were uniformly dispersed. Subsequently, cells were incubated at 37°C in a humidified atmosphere of 5% CO_2_ for 2 to 3 weeks until the colonies were visible to the naked eye. Then, the reaction is terminated and the cell suspension is abandoned. The cells were washed in PBS twice and fixed with 5 ml acetic acid/methanol (1 : 3 by vol). After 15 min of fixation, the acetic acid/methanol was removed and cells were stained with Giemsa for 10 to 30 min. After that, staining solution was washed away and the cell culture plate was dried. Cells placed in plate covered with transparent film were observed directly by naked eyes or counted under an optical microscope at low magnification for numbers of clones with more than 10 cells to determine the clone formation rate.

### 2.5. Cell Scratch Test

Cell scratch test was performed as described previously [15]. Briefly, the cells of control group and experimental group were plated in 6-well plates. When cells were grown to 90% fusion, a 100 *μ*L pipette tip was used to slightly draw three scratches in the plate. After that, cells were washed by PBS and incubated with serum-free medium. The gap was measured immediately after cell scratch and after 24 h of incubation under the low-magnification phase-contrast microscope (Olympus MK, Tokyo, Japan). Migration rate = (0 h scratch distance–24 h scratch distance)/0 h scratch distance.

### 2.6. Reverse Transcription-Polymerase Chain Reaction (RT-PCR) Analysis

U251 and U87 cells were firstly dissolved in 1 ml of Trizol (Thermo Fisher Scientific, MA, USA), and RNA was extracted according to Trizol instructions. After quantified, RNA was then reverse transcribed into cDNA. Further, PCR reaction system was configured according to fluorescence quantitative PCR kit (Takara, Dalian, China) instructions. The real-time quantitative RT-PCR assay was carried out by ABI7500 qPCR instrument (Applied Biosystems Inc., Foster City, CA, USA) with the reaction conditions of predenaturation at 95°C for 10 min, followed by 40 cycles of denaturation at 95°C for 10 s, annealing at 60°C for 20 s, and extension at 72°C for 34 s. Then, the expression levels of lncRNA-MEG3, miR-6088, SMARCB1, E-cadherin, N-cadherin, Vimentin, and Snail-1 were detected. RT-PCR primers are shown in Table 1. All primers were synthesized by GENEWIZ, Inc. (Beijing China). The internal reference of mRNA was GAPDH, and the internal reference of miRNA expression was U6. The data analysis was conducted by 2^−ΔΔCt^ method [16], with the following formula:

(1) $$\begin{array}{c} \Delta \Delta \text{Ct}={\left[{\text{Ct}}_{\left(\text{target gene}\right)}-{\text{Ct}}_{\left(\text{reference gene}\right)}\right]}_{\text{experimental group}}-{\left[{\text{Ct}}_{\left(\text{target gene}\right)}-{\text{Ct}}_{\left(\text{reference gene}\right)}\right]}_{\text{control group}} \end{array}$$

### 2.7. Western Blot Analysis

The U81 or U251 cells treated for 48 h were washed with precooled PBS buffer for 3 times and then added with protein extraction lysis buffer in a 100 *μ*L/50 mL culture flask on ice for 30 min. Subsequently, under the condition of 12000 rpm at 4°C, cells were centrifuged for 10 min. The collected supernatant was split into 0.5 mL centrifuge tubes and stored at −20°C or subjected to protein quantification using BCA kits (Wanlei Biological Technology Co., Shenyang, China). Next, 6 × SDS loading buffer was added at 100°C for protein denaturation. The protein was then separated by SDS electrophoresis, and the membrane was transferred by a precooled transfer buffer at 4°C for 1.5 h. The membrane was then blocked with 5% nonfat milk in TBST buffer for 1 h. TBST configured primary antibodies of E-cadherin (14472S, 1 : 100, Cell Signaling Technology, MA, USA), N-cadherin (13116S, 1 : 1000, Cell Signaling Technology, MA, USA), Vimentin (5741S, 1 : 1000, Cell Signaling Technology, MA, USA), SMARCB1 (ab222519, 1 : 1000, abcam, CA, USA), Snail-1 (ab53519, 1 : 500, abcam, CA, USA), and *β*-actin (ab4970s, 1 : 1000, abcam, CA, USA) were separately incubated with the membrane at 4°C overnight before TBST wash for 3 × 10 min. After that, goat anti-rabbit IgG or goat anti-mouse IgG (1 : 5000, Beijing ComWin Biotech Co., Ltd, China) was used for incubation at room temperature for 2 h, followed by TBST wash. Expression levels of proteins were determined after color development.

### 2.8. Dual-Luciferase Reporter Gene Assay

The target sites for binding of lncRNA-MEG3 and miR-6088 and for miR-6088 and SMARCB1 were predicted by the online software Starbase. According to the prediction, the binding sites of mutant sequences and wild sequences of MEG3 and miR-6088 and of SMARCB1 and miR-6088 were designed, respectively. The mutant and wild sequence fragments were cloned and conjugated to the Promega vector, designated MT-MEG3, WT-MEG3, MT-SMARCB1, and WT-SMARCB1, respectively, and then separately transfected with miR-6088 mimic or miR-6088 inhibitor into HEK-293T cells. After 48 h, fluorescence intensity of each group was measured using a luciferase kit (Beijing Yuanpinghao Biotechnology Co., Ltd.).

### 2.9. Statistical Analysis

Data were analyzed utilizing SPSS 18.0 (IBM Corp., Armonk, NY, USA) and GraphPad Prism 6.0 (GraphPad Software Inc.). Continuous data were expressed as mean ± standard deviation. Two groups were compared using *t*-test, and multiple groups were compared using one-way analysis of variance (ANOVA). *P* < 0.05 was identified as statistically significant.

## 3. Results

### 3.1. MEG8 and SMARCB1 Expressions Were Downregulated in Glioma Cells

**Figure 1:**
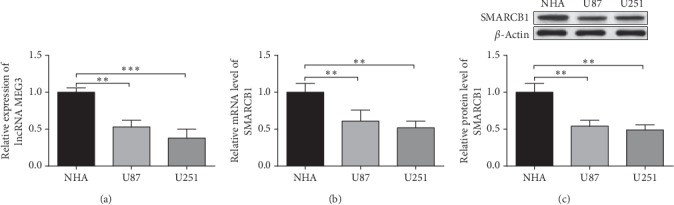
MEG3 and SMARCB1 are downregulated in glioma cells. Note: the detection of NHA, U87, and U251 cells, the expression levels of lncRNA MEG3 were measured by RT-PCR (a). The mRNA and protein expressions of SMARCB1 were detected by RT-PCR (b) and Western blot assay (c), respectively. ^*∗∗*^*P* < 0.01, ^*∗∗∗*^*P* < 0.001 versus NHA group; NHA, normal human astrocytes.

Quantitative PCR and Western blot showed that U87 and U251 cells had decreased lncRNA-MEG3 expression (*P* < 0.01) (Figure 1(a)), as well as the decreased mRNA and protein levels of SMARCB1 (*P* < 0.01) (Figures 1(b) and 1(c)) compared with NHA. The aberrant profile of lncRNA-MEG3 and SMARCB1 in glioma cells indicates their implication in glioma progression.

### 3.2. LncRNA-MEG3 Could Suppress Proliferation and Migration in Glioma Cells

**Figure 2:**
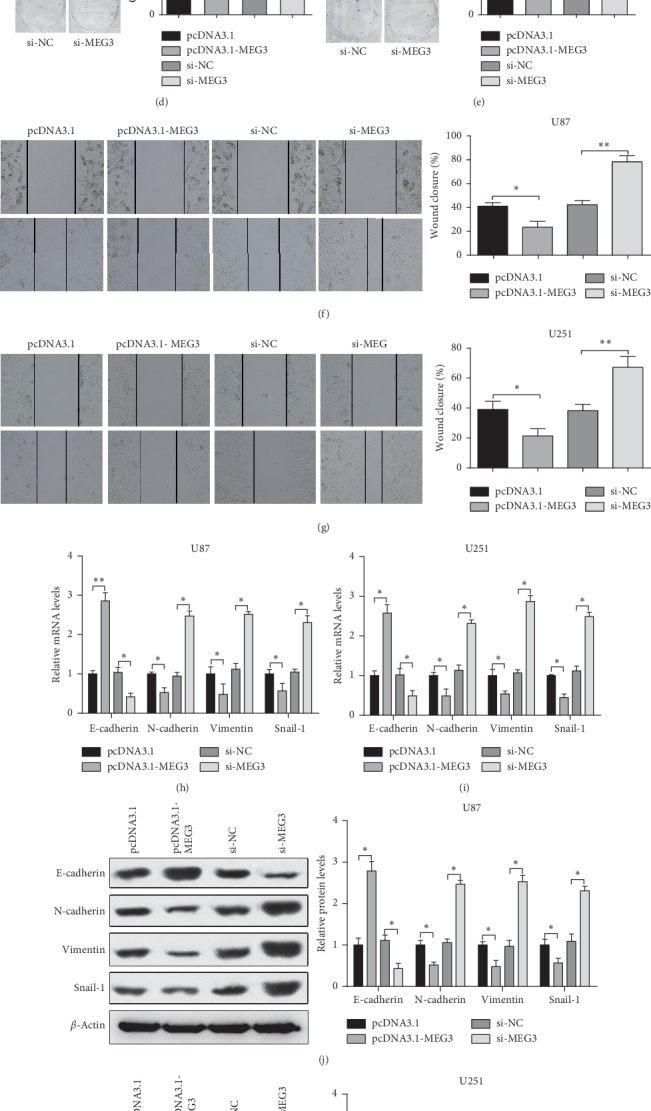
MEG3 on cell proliferation and migration of glioma cells. Note: after U87 and U251 cells were transfected with pcDNA3.1, pcDNA3.1-MEG3, si-NC, or si-MEG3 plasmid, mRNA of MEG3 was detected by RT-PCR (a). Proliferation ability of U87 or U251 cells was measured by MTT assay (b-c). The number of clones of U87 or U251 cells was determined by colony formation assay (d-e). Cell migration ability of U87 or U251 cells was tested by cell scratch test (f-g). The mRNA and protein expressions of EMT (E-cadherin, N-cadherin, Vimentin, and Snail-1) in U87 or U251 cells were measured by RT-PCR (h-i) and Western blot (j-k), respectively. ^*∗*^*P* < 0.05, ^*∗∗*^*P* < 0.01; MEG3, maternally expressed gene 3; EMT, epithelial-mesenchymal transition.

To determine the role of MEG3 in the development of glioma cells, glioma cell lines were transfected with pcDNA3.1-MEG3 or MEG3 inhibitor. Compared with pcDNA3.1 group, the expression of MEG3 in the pcDNA3.1-MEG3 group was clearly increased, while it was significantly reduced in the MEG3 inhibitor group in comparison with that in the inhibitor NC group (*P* < 0.01) (Figure 2(a)), which indicated high transfection efficiency of MEG3 overexpression and silencing in U87 and U251 cells.

Next, we measured the effect of MEG3 overexpression and silencing on cell proliferation using MTT and colony formation experiment in U87 and U251 cells. The results showed that cell proliferation and cell clones were markedly decreased in the pcDNA3.1-MEG3 group, compared with the pcDNA3.1 group. However, transfection of si-MEG3 could increase cell proliferation and cell clones (si-MEG3 group versus si-NC group) (*P* < 0.01) (Figures 2(b)–2(e)). Taken together, MEG3 could repress cell proliferation of U81 and U251 cells.

Subsequently, cell scratch assay confirmed that, compared with the pcDNA3.1 group, the cell migration ability of cells in the pcDNA3.1-MEG3 group was weakened, while it was enhanced in the si-MEG3 group compared with the si-NC group (*P* < 0.01) (Figures 2(f) and 2(g)).

Then, the expressions of epithelial-mesenchymal transition (EMT) related markers such as E-cadherin, N-cadherin, and Vimentin, as well as the transcription factor Snail-1, were measured by RT-PCR and Western blot assay. The results showed that, compared with the pcDNA3.1 group, E-cadherin protein and mRNA expression levels were distinctly increased, while protein and mRNA expression levels of N-cadherin, Vimentin, and Snail-1 were markedly reduced in the pcDNA3.1-MEG3 group. However, reversed expression patterns were found in the si-MEG3 group (*P* < 0.01) (Figures 2(h)–2(k)). Taken together, MEG3 inhibits cell migration and EMT of glioma cells.

### 3.3. SMARCB1 Could Hinder Proliferation and Migration in Glioma Cells

**Figure 3:**
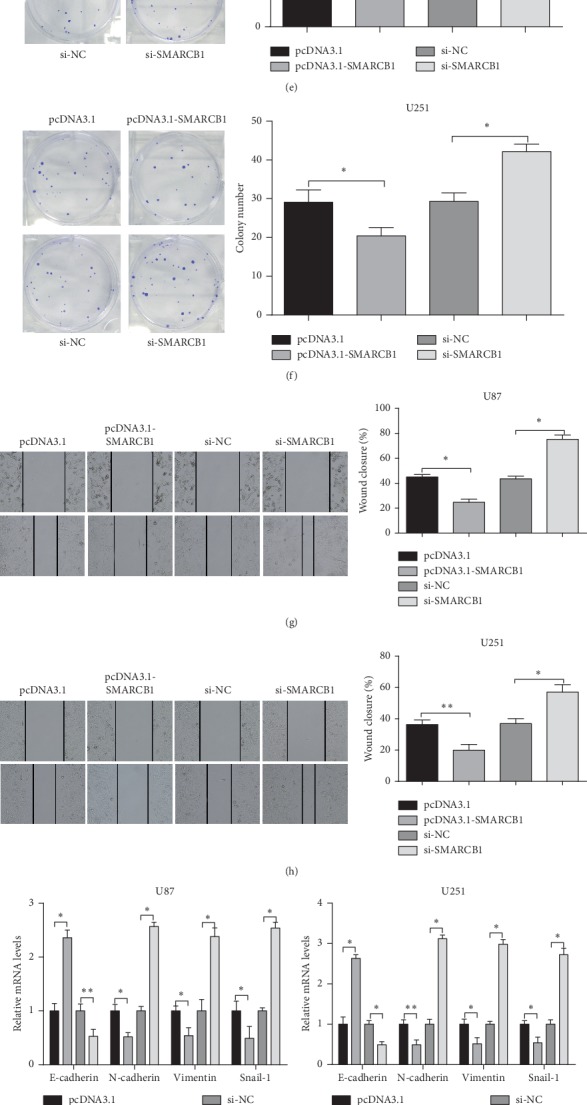
SMARCB1 on cell proliferation and migration of glioma cells. Note: after U87 and U251 cells were transfected with pcDNA3.1, pcDNA3.1-SMARCB1, si-NC, or si-SMARCB1 plasmid, the mRNA and protein expressions of SMARCB1 were inspected by RT-PCR (a) and Western blot (b), respectively. MTT assay was used to estimate the cell proliferation ability of U87 (c) or U251 (d) cells. The number of clones of U87 or U251 cells was determined by colony formation assay (e-f). Cell migration ability of U87 or U251 cells was tested by cell scratch test (g-h). The mRNA and protein expressions of EMT (E-cadherin, N-cadherin, Vimentin, and Snail-1) in U87 or U251 cells were measured by RT-PCR (i-j) and Western blot (k-l), respectively. ^*∗*^*P* < 0.05, ^*∗∗*^*P* < 0.01; EMT, epithelial-mesenchymal transition.

This study further investigated the function of SMARCB1 on glioma cells. For this reason, the effects of the overexpression and inhibition of SMARCB1 in U87 and U251 cells were detected by RT-PCR and Western blot assay. Compared with the pcDNA3.1 group, SMARCB1 protein and mRNA expression levels were distinctly increased in the pcDNA3.1-SMARCB1 group, while it was clearly decreased in the SMARCB1 inhibitor group in comparison with that in the inhibitor NC group (*P* < 0.01) (Figures 3(a) and 3(b)), indicating that the cell transfection achieved high transfection efficiency of SMARCB1 overexpression and silencing in glioma cells.

Moreover, cell proliferation and cell clones were measured in U251 and U87 cells. The detection showed that cell proliferation and colony formation ability were markedly decreased in the pcDNA3.1-SMARCB1 group, compared with the pcDNA3.1 group. However, transfection of si-SMARCB1 could increase cell proliferation and cell clones (si-SMARCB1 group versus si-NC group) (*P* < 0.01) (Figures 3(c)–3(f)). Taken together, SMARCB1 could inhibit proliferation of U81 and U251 cells.

Subsequently, cell scratch assay demonstrated that, compared with the pcDNA3.1 group, the cell migration ability of cells in the pcDNA3.1-SMARCB1 group was weakened, while it was enhanced in the si-SMARCB1 group compared with the si-NC group (*P* < 0.01) (Figures 3(g) and 3(h)).

Then, the expressions of EMT-related markers were measured by RT-PCR and Western blot assay, which showed that, compared with the pcDNA3.1 group, E-cadherin protein and mRNA expression levels were distinctly increased, while mRNA and protein expression levels of N-cadherin, Vimentin, and Snail-1 were decreased markedly in the pcDNA3.1-SMARCB1 group. However, reversed expression patterns were found in the si-SMARCB1 group (*P* < 0.01) (Figures 3(i)–3(l)). Herein, SMARCB1 inhibits cell migration and EMT of glioma cells.

### 3.4. MEG3 Negatively Targets miR-6088 to Regulate SMARCB1

**Figure 4:**
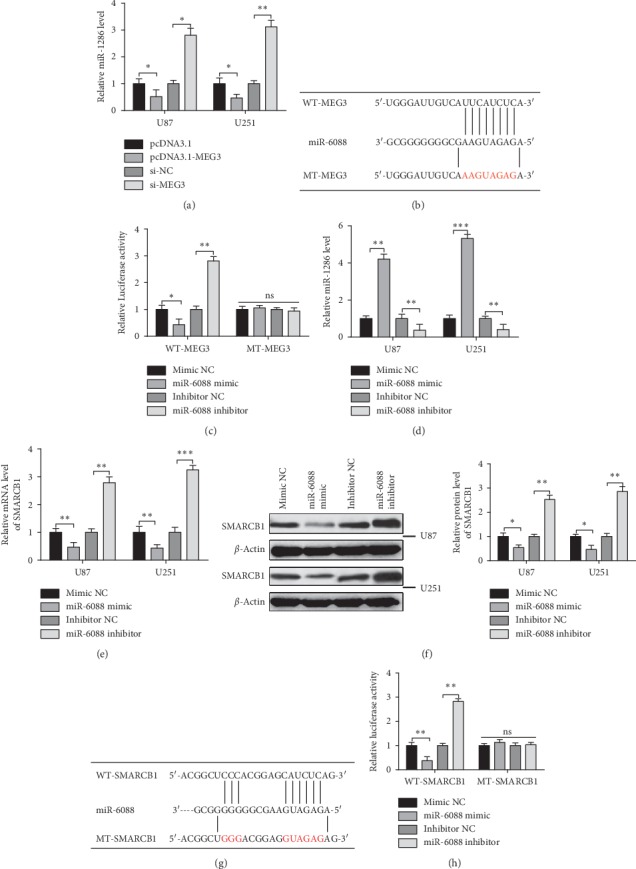
MEG3 interacts with miR-6088 to regulate SMARCB1. Note: pcDNA3.1, pcDNA3.1-MEG3, si-NC, or si-MEG3 plasmid was transfected into U87 and U251 cells. RT-PCR was applied to detect miR-6088 expression (a). Starbase predicted the binding sites of MEG3 and miR-6088, and the mutation sites of MEG3 were designed (b). HEK-293T cells were transfected or cotransfected with mimic NC, miR-6088 mimic, inhibitor NC, or miR-6088 inhibitor with WT-SMARCB1 or MT-SMARCB1. Fluorescent activity was surveyed by dual-luciferase reporter gene (c). Mimic NC, miR-6088 mimic, inhibitor NC, or miR-6088 inhibitor was transfected into U87 and U251 cells, and qRT-PCR was applied to detect the transfection efficiency of the overexpression and inhibition of miR-6088 (d) in U87 and U251 cells. SMARCB1 mRNA (e) and protein expression levels (f) were surveyed by RT-PCR and Western blot, respectively. Starbase predicted the binding site of miR-6088 and SMARCB1. The mutation site of SMARCB1 was designed (g). HEK-293T cells were transfected or cotransfected with mimic NC, miR-6088 mimic, inhibitor NC, or miR-6088 inhibitor with WT-SMARCB1 or MT-SMARCB1. Fluorescent activity was surveyed by dual-luciferase reporter gene assay (h). ^*∗*^*P* < 0.05, ^*∗∗*^*P* < 0.01, ^*∗∗∗*^*P* < 0.001.

As we proved that both MEG3 and SMARCB1 are implicated in glioma cells, no direct interaction is found between MEG3 and SMARCB1. Therefore, we conjectured that MEG3 may regulate certain intermediate to affect SMARCB1 in glioma cells. DIANA and Starbase showed possible binding sites between MEG3 and miR-6088 as well as miR-6088 and SMARCB1. In this regard, we further reasoned that MEG3 mediates miR-6088, which in turn regulates SMARCB1 in glioma cells. To testify this hypothesis, the overexpression and silencing of MEG3 were surveyed in U251 and U87 cells. RT-PCR results manifested that, compared with the pcDNA3.1 group, miR-6088 expressions in the pcDNA3.1-MEG3 group were clearly reduced while miR-6088 expressions in the si-MEG3 group showed opposite tendency in comparison with the si-NC group (*P* < 0.05) (Figure 4(a)). The predicated binding sites and designed mutation sites of MEG3 and miR-6088 are shown in Figure 4(b). To confirm the targeting relationship regarding MEG3 and miR-6088, we constructed a wild-type MEG3 luciferase promoter plasmid (designated WT-MEG3) and a mutant MEG3 luciferase promoter plasmid (designated MT-MEG3) containing 6 mutation sites predicted to bind to miR-6088. Furthermore, the detection of MEG3 promoter luciferase reporter gene showed that, compared to the control group, the fluorescence activity was significantly decreased after cells were cotransfected with WT-MEG3 and miR-6088 mimic (*P* < 0.01). When WT-MEG3 was cotransfected with miR-6088 inhibitor, the fluorescence activity was markedly increased (*P* < 0.01). There was no obvious difference in fluorescence activity in response to the cotransfection of MT-MEG3 with miR-6088 mimic or miR-6088 inhibitor (Figure 4(c)). These experiments indicated that MEG3 binds directly to miR-6088.

Meanwhile, miR-6088 was also induced to be overexpressed or inhibited in U87 and U251 cells. The results uncovered that, compared with the mimic NC group, miR-6088 mimic group was found with elevated miR-6088 expression and decreased SMARCB1 expression. However, reversed expression patterns were displayed in the miR-6088 inhibitor group (*P* < 0.05) (Figures 4(d)–4(f)). Altogether, these results indicated that miR-6088 negatively regulated SMARCB1. The binding and mutant sites of SMARCB1 3′-UTR and miR-6088 predicated by Starbase are shown in Figure 4(g). To confirm their targeting relationship, we constructed a wild-type SMARCB1 3′-UTR luciferase promoter plasmid (designated WT-SMARCB1) and a mutant SMARCB1 3′-UTR luciferase promoter plasmid (designated MT-SMARCB1) containing 10 mutation sites predicted to bind to miR-6088. Furthermore, the detection of SMARCB1 promoter luciferase reporter gene with the results showed that, compared to the control group, the fluorescence activity was significantly lower after cells were cotransfected with WT-SMARCB1 and miR-6088 mimic (*P* < 0.01). When WT-SMARCB1 was cotransfected with miR-6088 inhibitor, the fluorescence activity was markedly increased (*P* < 0.01). There was no obvious difference in fluorescence activity in response to the cotransfection of MT-SMARCB1 with miR-6088 mimic or miR-6088 inhibitor (Figure 4(h)). These findings indicated that SMARCB1 binds directly to miR-6088.

### 3.5. MEG3 Blocks Proliferation and Migration in Glioma Cells via miR-6088/SMARCB1 Axis

**Figure 5:**
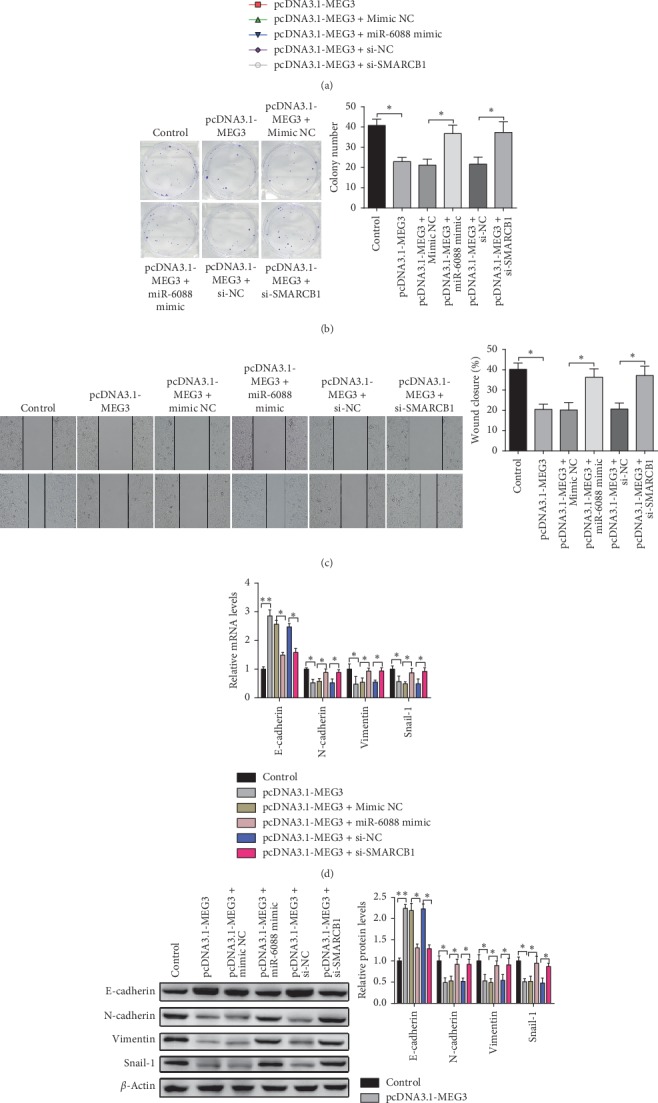
MEG3 participates in glioma progression via miR-6088/SMARCB1 axis. Note: after pcDNA3.1-MEG3, miR-6088 mimic, or si-SMARCB1 and their negative controls were transfected or cotransfected into U251 cells, cell proliferation ability, number of clones, and wound healing ability were separately measured by MTT assay (a), colony formation assay (b), and cell scratch test (c). The mRNA and protein expressions of EMT (E-cadherin, N-cadherin, Vimentin, and Snail-1) were determined by RT-PCR (d) and Western blot (e), respectively. ^*∗*^*P* < 0.05, ^*∗∗*^*P* < 0.01; EMT, epithelial-mesenchymal transition.

To verify whether there is a ceRNA mechanism of MEG3 in the development of glioma cells, pcDNA3.1-MEG3, miR-6088 mimic, or/and si-SMARCB1 were transfected into U251 cells. The results manifested that the cell proliferation and cell clones in the pcDNA3.1-MEG3 group were distinctly decreased when compared to the control group. Meanwhile, reversed expression patterns were found in the pcDNA3.1-MEG3 + miR-6088 mimic and pcDNA3.1-MEG3 + si-SMARCB1 groups in comparison with those in the pcDNA3.1-MEG3 + mimic NC and pcDNA3.1-MEG3 + si-NC groups, respectively (*P* < 0.01) (Figures 5(a) and 5(b)). Altogether, these findings indicate that the overexpression of MEG3 can inhibit glioma cell progression, while the overexpression of miR-6088 or the inhibition of SMARCB1 can partially reverse the inhibitive effect of pcDNA3.1-MEG3 on glioma cells.

Moreover, cell scratch assay revealed that, compared to the control group, cell migration ability in the pcDNA3.1-MEG3 group was suppressed while cell migration ability of the pcDNA3.1-MEG3 + miR-6088 mimic and pcDNA3.1-MEG3 + si-SMARCB groups was enhanced in comparison with its negative control group (*P* < 0.01) (Figure 5(c)). EMT-related markers were measured by RT-PCR and Western blot assay, which presented that protein and mRNA expression levels of E-cadherin in the pcDNA3.1-MEG3 group were obviously elevated while protein and mRNA expression levels of N-cadherin, Vimentin, and Snail-1 were distinctly reduced in comparison with the control group. However, protein and mRNA expression levels of E-cadherin in the pcDNA3.1-MEG3 + miR-6088 mimic and pcDNA3.1-MEG3 + si-SMARCB groups were obviously decreased while mRNA and protein expression levels of N-cadherin, Vimentin, and Snail-1 were distinctly increased in comparison with its negative control group (*P* < 0.01) (Figures 5(d) and 5(e)). Altogether, these results revealed that miR-6088 mimic and SMARCB1 silencing could partially rescue pcDNA3.1-MEG3 induced EMT and migration of glioma cells. Collectively, MEG3 can hinder cell migration and proliferation of glioma cells via modulating miR-6088/SMARCB1 axis.

## 4. Discussion

Glioma has been identified as one of the most aggressive primary tumors discovered in recent years with limited treatment options [17]. Therefore, the investigation on the molecular mechanism of glioma cells and the exploration of the effective treatment methods are essential to improve its unfavorable outcomes. In the present work, we uncovered that lncRNA MEG3 was obviously downexpressed in glioma cells, and we revealed that lncRNA MEG3 can function as a ceRNA to mediate miR-6088/SMARCB axis and thereby decrease cell migration, proliferation, and EMT in glioma cells.

LncRNAs play both physiological and pathological roles in cells [18]. In recent years, lncRNAs are described as critical regulators of tumor biology in glioma [19]. Here, largely depressed MEG3 was identified in U87 and U251 cells in comparison with the normal cells. In our finding, we assured that MEG3 blocked the proliferation, invasion, and migration of glioma cells, which was in line with previously published data [7, 11, 20]. Moreover, it has been reported that cancer cells can be mechanically guided by EMT processes, which play key roles in the invasion, migration, and metastasis of multiple malignancies [21]. EMT, known as epithelial to mesenchymal transformation, could increase individual cell motility [22]. In our study, we initially aimed to recognize the potential and novel markers, especially lncRNA, which affected the EMT progression in glioma cells. This result was in keeping with a previous work which confirmed that MEG3 inhibited cell invasion and EMT in breast cancer [23]. In view of cells that have undergone EMT, namely, acquiring resistance to senescence and even to apoptosis [24], the suppression of MEG3 on EMT indicates the protective role of MEG3 in glioma cells by promoting cell apoptosis of tumor cells. Although these results were solid evidence to support the tumor suppressor role of MEG3 in glioma, the specific mechanism, however, is far from being illustrated.

Accumulating evidence has highlighted the important roles of lncRNAs acting as ceRNAs in cancer and tumor progression [25, 26]. The ceRNAs are frequently characterized as transcripts that are posttranscriptionally cross-regulated by competing for shared binding miRNAs. For example, lncRNA MEG3 by acting as a ceRNA against miR-181 could modulate gastric cancer progression, which may serve as an underlying target for antineoplastic therapies [27]. Hence, we further focused on the explorations of potential ceRNA network underlying MEG3 in the current study. Through bioinformatic analysis, we indicated that miR-6088 might be a direct target of MEG3. Interestingly, this study revealed that miR-6088 acted as a master miRNA involving in EMT of glioma cells. Here, miR-6088 silence partially reversed MEG3 knockdown mediated repression of migration and invasion, and even EMT process of glioma cells. For the above observations, we concluded that MEG3 can function as a ceRNA via sponging miR-6088 in glioma cells.

Relevant study has disclosed the interactions among lncRNA, miRNA, and target genes in the study of tumor mechanism. For instance, lncRNA MEG3 could hinder prostate cancer progression via modulating miR-9-5p/QKI-5 axis [28]. LncRNA MEG3 blocks cell proliferation and induces cell apoptosis in laryngeal cancer through regulating miR-23a/APAF-1 axis [29]. The downstream target gene of miR-6088 in our study was also identified. SMARCB1 is a tumor inhibitor gene located at 22q11.23 [30]. A previous study demonstrated that the downregulation of SMARCB1 expression is associated with the upregulation of miR-193a-5p and miR-671-5p expressions in pediatric chordomas [31]. Consistently, our findings uncover that SMARCB1 is downregulated in glioma cells and contributed to the suppression of cell migration and proliferation of glioma cells. SMARCB1/INI1 and key proteins in a variety of pathways, such as polycomb pathway, the p16-RB pathway, sonic hedgehog, and WNT signaling pathway, connected with tumor proliferation and progression [32]. This study identified that miR-6088 negatively targets SMARCB1. Herein, we can draw a conclusion that MEG3 regulates cell growth of glioma cells through mediating the miR-6088/SMARCB1 axis.

## 5. Conclusion

To conclude, this study recognizes lncRNA MEG3 as a tumor inhibitor and a potential therapeutic biomarker for glioma. The possible mechanism herein may involve the miR-6088/SMARCB1 axis. Nevertheless, more elaborated studies are needed to verify the results of our experiments. The proposal of the possible mechanism of the miR-6088/SMARCB axis may contribute to a better understanding of lncRNA MEG3 on glioma cells and facilitate the potential therapy for amelioration of the risk of glioma.

## Figures and Tables

**Table 1:** Primer sequence for quantitative reverse transcription-polymerase chain reaction to determine the expressions of E-cadherin, N-cadherin, Vimentin, Snail-1, MEG3, miR-6088, SMARCB1, GAPDH, and U6.

Name of primer	Sequences (5′-3′)
E-cadherin-F	CGTCGAGCTCTTGACCGAAA
E-cadherin-R	TCAAACACCTCCTGTCCTCT
N-cadherin-F	AGGGGAGAGGTGCTCTACTG
N-cadherin-R	GGGGTAATCCACACCACCTG
Vimentin-F	TCCGCACATTCGAGCAAAGA
Vimentin-R	TGAGGGCTCCTAGCGGTTTA
Snail-1-F	CGAGCCATAGAACTAAAGCC
Snail-1-R	TGAGGGAGGTAGGGAAGTG
MEG3-F	GACACCCTGCACCTATTCCC
MEG3-R	CAACAGCCCTGTGAGGTAGG
miR-6088-F	TCTTGCGGGGGGGCGAAG
miR-6088-R	CAGTGCGTGTCGTGGAGT
SMARCB1-F	TGTAAAACGACGACGGCCAGT
SMARCB1-R	CAGGAAACAGCTATGACC
GAPDH-F	ACCACAGTCCATGCCATCAC
GAPDH-R	TCCACCACCCTGTTGCTGTA
U6-F	TCGCTTCGGCAGCACATATAC
U6-R	GCGTGTCATCCTTGCGCAG

Note: F, forward; R, reverse.

## Data Availability

The datasets used or analyzed during the current study are available from the corresponding author upon request.
